# High Levels of Diversity in *Anopheles* Subgenus *Kerteszia* Revealed by Species Delimitation Analyses

**DOI:** 10.3390/genes14020344

**Published:** 2023-01-28

**Authors:** Brian P. Bourke, Richard C. Wilkerson, Fredy Ruiz-Lopez, Silvia A. Justi, David B. Pecor, Martha L. Quinones, Juan-Carlos Navarro, Joubert Alarcón Ormaza, Joubert Alarcón Ormaza, Ranulfo González, Carmen Flores-Mendoza, Fanny Castro, Jesús E. Escovar, Yvonne-Marie Linton

**Affiliations:** 1 Walter Reed Biosystematics Unit, Museum Support Center MRC-534, Smithsonian Institution, 4210 Silver Hill Rd., Suitland, MD 20746, USA; 2 One Health Branch, Walter Reed Army Institute of Research, 503 Robert Grant Ave., Silver Spring, MD 20910, USA; 3 Department of Entomology, Smithsonian Institution—National Museum of Natural History, 10th St NE & Constitution Ave NE, Washington, DC 20002, USA; 4 Program for the Study and Control of Tropical Diseases, Faculty of Medicine, University of Antioquia, Medellín 050010, Colombia; 5 Departamento de Salud Pública, Universidad Nacional de Colombia, Bogotá 111321, Colombia; 6 Research Group of Emerging and Neglected Diseases and Ecoepidemiology, Faculty of Health Science, Universidad Internacional SEK, Quito 170134, Ecuador; 7 Instituto de Ecologia y Zoologia Tropical, Facultad de Ciencias, Universidad Central de Venezuela, Caracas 1053, Venezuela; 8 Universidad Católica Santiago de Guayaquil, Guayaquil 090615, Ecuador; 9 Facultad de Ciencias Naturales y Exactas, Universidad del Valle, Calle 13 # 100-00, Ed 320, Cali 760032, Colombia; 10 Entomology Department, U.S. Naval Medical Research Unit. No 6, Bellavista, Lima APO AA 34031, Peru; 11 Escuela de Ciencias Básicas y Aplicadas, Universidad de La Salle, Bogotá 111001, Colombia

**Keywords:** DNA barcoding, *Kerteszia*, species delimitation, neivai complex, laneanus complex, bellator complex, boliviensis complex, homunculus complex, rollai complex, pholidotus complex

## Abstract

The *Anopheles* subgenus *Kerteszia* is a poorly understood group of mosquitoes that includes several species of medical importance. Although there are currently twelve recognized species in the subgenus, previous studies have shown that this is likely to be an underestimate of species diversity. Here, we undertake a baseline study of species delimitation using the barcode region of the mtDNA *COI* gene to explore species diversity among a geographically and taxonomically diverse range of *Kerteszia* specimens. Beginning with 10 of 12 morphologically identified *Kerteszia* species spanning eight countries, species delimitation analyses indicated a high degree of cryptic diversity. Overall, our analyses found support for at least 28 species clusters within the subgenus *Kerteszia*. The most diverse taxon was *Anopheles neivai*, a known malaria vector, with eight species clusters. Five other species taxa showed strong signatures of species complex structure, among them *Anopheles bellator*, which is also considered a malaria vector. There was some evidence for species structure within *An. homunculus*, although the results were equivocal across delimitation analyses. The current study, therefore, suggests that species diversity within the subgenus *Kerteszia* has been grossly underestimated. Further work will be required to build on this molecular characterization of species diversity and will rely on genomic level approaches and additional morphological data to test these species hypotheses.

## 1. Introduction

Malaria remains one of the most important infectious human diseases globally. In 2020 alone, there were an estimated 241 million malaria cases across 85 countries [1]. Although the great majority of these cases (>95%) are in Africa, malaria remains a major health concern in the Americas. In recent years, progress in reducing the malaria burden in South America has stalled and malaria is resurgent, with annual case numbers reaching over 1 million [2,3]. The Amazon basin, with its tropical forests, remains the epicenter of malaria transmission [2,4], and it is also an important source for extra-Amazonian malaria transmission [5]. 

One of the more important regions for autochthonous cases of extra-Amazonian malaria in South America is southeast Brazil, particularly the Atlantic Forest. Here, low-level *Plasmodium* parasite transmission is associated with local non-human primates serving as reservoirs and *Kerteszia* mosquitoes as vectors, as opposed to mainly those *Anopheles* in the subgenus *Nyssorhynchus* mosquitoes in the Amazon [6]. Several *Kertezsia* species are currently considered vectors of *Plasmodium* and, due to their selection of epiphytic bromeliads as larval habitats, malaria cases associated with these vectors have become known as “bromeliad malaria” [7,8,9]. Malaria epidemics in southeast Brazil during the 19th and early 20th century were largely attributed to bromeliad malaria, and the resulting control measures implemented in the 1940s relied heavily on deforestation to remove the bromeliad larval breeding habitat [10]. Malaria epidemics were the major public health concern in Trinidad up to the early 20th Century, and transmission on agricultural land was largely attributed to bromeliad malaria [11,12].

There are currently 12 formally described species within the subgenus *Kertezsia*, which includes: *Anopheles auyantepuiensis*, Harbach & Navarro, 1996; *An*. *bambusicolus*, Komp, 1937; *An*. *bellator*, Dyar & Knab, 1906; *An*. *boliviensis*, Theobald, 1905; *An*. *cruzii*, Dyar & Knab, 1908; *An*. *gonzalezrinconesi*, Cova García, Pulido F. & Escalante de Ugueto, 1977; *An*. *homunculus*, Komp, 1937; *An*. *laneanus*, Corrêa & Cerqueira, 1944; *An*. *lepidotus*, Zavortink, 1973; *An*. *neivai*, Howard, Dyar & Knab, 1912; *An*. *pholidotus*, Zavortink, 1973; and *An*. *rollai*, Cova García, Pulido F. & Escalante de Ugueto, 1977 [13]. *Kertezsia* mosquitoes are widely distributed from southern Mexico to northern Argentina [13]. Four species are considered malaria vectors: *An. bellator* [14], *An. cruzii* [15], *An. homunculus* [14], and *An. neivai* [16] and there is emerging evidence that *An*. *cruzii*, *An. neivai* [17,18,19,20], and *An*. *homunculus* [21] comprise cryptic species complexes.

Detecting and delimiting cryptic species diversity is fundamentally important to understanding ecology and life history strategy, vector identification and incrimination, and ultimately, vector management and disease control. The current study aims to provide a baseline description of species diversity in the subgenus *Kertezsia* by analyzing cytochrome c oxidase I (*COI*) barcode data using species delimitation approaches [22,23,24]. 

## 2. Materials and Methods

### 2.1. Taxon Sampling

A total of 272 specimens, representing 10 of the 12 formally recognized species of *Anopheles Kerteszia,* collected over 30 years from across Central and South America, were included in the current analyses. Newly generated mtDNA *COI* barcode sequences and associated data, along with publicly available sequences, are available on the Barcode of Life Database (BOLD) under Dataset: DS-KERT. This dataset comprised *An*. *bambusicolus* (*n* = 3), *An*. *bellator* (*n* = 14), *An*. *boliviensis* (*n* = 10), *An*. *cruzii* (*n* = 62), *An*. *homunculus* (*n* = 17), *An*. *laneanus* (*n* = 8), *An*. *lepidotus* (*n* = 7), *An*. *neivai* (*n* = 107), *An*. *pholidotus* (*n* = 10), and *An*. *rollai* (*n* = 23) from Brazil, Colombia, Ecuador, French Guiana, Trinidad and Tobago, Panama, and Venezuela. In addition, an unidentified *Kerteszia* specimen (NAMRU6_2014_342) from Peru and ten further “Culicidae spp.” sequences closely related to *Kerteszia* using NCBI Blast alignment, were also included. Verified specimens of *An*. *auyantepuiensis* and *An*. *gonzalezrinconesi* were unavailable for analyses. All specimens were morphologically identified using available morphological keys and original species descriptions [25,26,27]. Morphological voucher specimens were retained where available, and included point-mounted adults of both sexes, slide-mounted associates of male genitalia, and immature stages (larval exuviae, Le; pupal exuviae, Pe). Extracted DNA was retained in frozen repositories of the National Museum of Natural History, Smithsonian Institution, USA (USNM), and the Natural History Museum, England (NHMUK). 

### 2.2. DNA Barcodes

Specimens for this study were accrued by members of the Mosquito Barcoding Initiative (MBI). Legs and/or abdomens from 90 vouchered morphologically identified specimens were used for DNA extraction following the phenol–chloroform method, using the AutoGen or QIAgen BioSprint automated extraction platform. Amplification of the COI barcode region (658-bp) was performed using the Folmer LCO1490 and HCO2198 primers [28] and the PCR conditions described in Ruiz et al. [29]. Sequencing was carried out in both directions using the Big Dye^®^ Terminator Kit on an ABI 3730 automated sequencer (PE Applied Biosystems). Sequences were edited in Sequencher^TM^ v. 4.8 (Genes Codes Corporation, Ann Arbor, MI, USA). An additional 182 mtDNA COI sequences, publicly available in the GenBank (www.ncbi.nlm.nih.gov/genbank; accessed on 10 October 2022) and BOLD (www.boldsystems.org; accessed on 10 October 2022) databases, were included in the study (Supplementary Table S1). The full sequence dataset was then aligned, first by nucleotides using the Muscle algorithm [30] implemented in SeaView [31], and then by amino acid using TranslatorX [32]. 

### 2.3. Exploratory Data Analysis

The NeighborNet algorithm implemented in SplitsTree [33] was used to construct a network to explore phenetic relationships and assess whether the data nature was tree-like, with a bifurcating phylogeny, or network-like, with reticulation and conflicting phylogenetic signals. 

### 2.4. Phylogeny

A 70% majority-rule consensus tree was constructed by maximum likelihood using 1000 replicates from the ultrafast bootstrap approximation approach (UFBoot) implemented in IQ-TREE 2 [34]. The UFBoot support values are more unbiased and support for a clade should be considered at ≥95%. Data were partitioned by codon position and optimal model selection was performed using the -m MFP option among a range of models (-mset mrbayes). The selection of an appropriate outgroup followed the recommendations of Grant [35], by including the closest known sister taxa and successively expanding the outgroup sample until ingroup topology was shown to be stable in at least two iterations. Outgroup sampling included specimens from the following subgenera: *Nyssorhynchus* (*An. albitarsis*, MF381591; *An. oryzalimnetes*, HQ335345; *An. darlingi*, GQ918272; *An. evansae*, MF381711; *An. nuneztovari*, MF381680; *An. strodei*, NC037808; *An. braziliensis*, NC037791); *Anopheles* (*An. minor*, MF381684); and *Stethomyia* (*An. kompi,* NC037827; *An. nimbus*, NC037811). 

### 2.5. Species Delimitation

The first method used for the species delimitation analyses was the Assemble Species by Automatic Partitioning (ASAP [23]), which uses pairwise genetic distances and ascending hierarchical clustering to build a list of best partitions. Partitions are ranked by score, which is a combination of two metrics: the probability of panmixia and the barcode gap width. The method does not require any *a priori* knowledge of species number/composition, or biological information, such as a phylogeny or intraspecific distances. We also used the Barcode of Life Database (BOLD: www.boldsystems.org; accessed on 10 October 2022) Barcode Index Numbers (BINs). BOLD BINs are produced from the Refined Single Linkage (RESL) analysis of the entire BOLD database, which clusters sequences into operational taxonomic units (OTUs) using a graph analytical approach [24]. However, not all specimens/clusters had been assigned BOLD BIN numbers at the time of manuscript submission (11 from 82 specimens of *An. neivai* s.s.), due to delays associated with the automated BIN analysis running on BOLD. The Multi-rate Poisson tree processes (mPTP [22]) approach was also used. This approach is a phylogeny-aware delimitation method that uses differences in mutation rate in a phylogenetic tree to distinguish interspecific and intraspecific diversity. Markov Chain Monte Carlo (MCMC) was used to assess confidence, using 4 independent runs. 

## 3. Results

### 3.1. Phylogenetic Network and Tree

**Figure 1:**
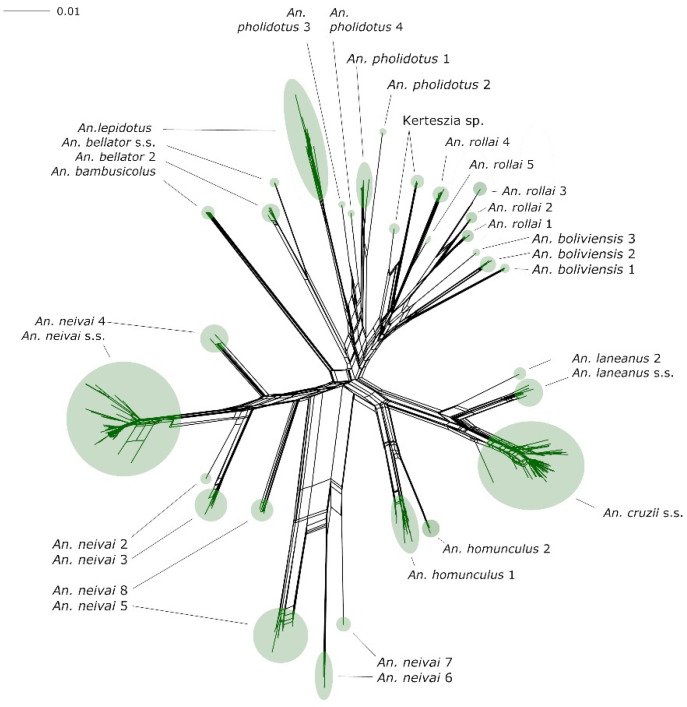
SplitsTree (NeighborNet) network analysis of the subgenus *Kerteszia* using the *COI* barcode, showing the tree-like nature of the data at internal positions.

**Figure 2:**
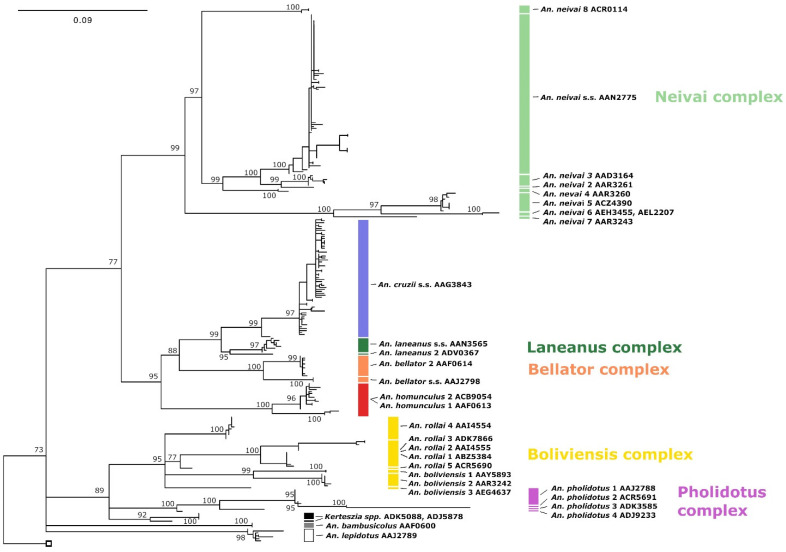
Maximum likelihood (70% majority-rule bootstrap consensus) *COI* barcode tree of the subgenus *Kerteszia*. The UFBoot approximation is significant at the level of 95% support.

The phylogenetic network demonstrates the tree-like versus network-like nature of the data, as well as the clustering of sequences into tentative species (Figure 1). As can be seen, there is a strong network-like structure among the deeper phylogenetic relationships, while the relationships among the tips and at the level of species tend to be bifurcating and more tree-like. These relationships are also consistent with the phylogenetic tree (Figure 2); all species and species complex clades are supported by the ultrafast bootstrap approximation at values ≥95%. However, relationships between the members of complexes and those found at more basal positions are all poorly supported or resolved. 

### 3.2. Species Delimitation

The species delimitation using the distance-based ASAP approach yielded 28 putative species (vertical bars in Figure 2). BOLD BINs were associated with all clusters delimited by ASAP. In some cases, multiple BINs were detected per ASAP cluster (BOLD BINs associated with taxon names in Figure 2). The mPTP delimitation was not included because the phylogenetic tree, upon which mPTP relies, was poorly supported, and the SplitsTree network suggested that the data were not strongly tree-like. 

Species such as *An. lepidotus* (BOLD:AAJ2789), *An. bambusicolus* (BOLD:AAF0600), and *An. cruzii* (BOLD:AAG3843) are well-resolved taxa in the phylogenetic tree (≥98% bs support) and are separately clustered in the species delimitation analyses. However, all other taxa in the genus show at least some evidence of species complex structure.

*Anopheles pholidotus* is split into four clusters—*An. pholidotus* 1 (BOLD:AAJ2788), *An. pholidotus* 2 (BOLD:ACR5691), *An. pholidotus* 3 (BOLD:ADK3585), and *An. pholidotus* 4 (BOLD:ADJ9233)—with minimum inter-cluster distances of between 2.92% and 7.03% (K2P distance). None of the specimens were collected near the type locality in northern Panama, and so none of the clusters could be identified as a topotype (Table 1).

Both *An. boliviensis* and *An. rollai* show evidence of species complex status. *Anopheles boliviensis* is delimited into three clusters (*An. boliviensis* 1, BOLD:AAY5893; *An. boliviensis* 2, BOLD:AAR3242; *An. boliviensis* 3, BOLD:AEG4637), with minimum inter-cluster distances of 4.31–5.34%. The ASAP analysis splits *Anopheles rollai* into three clusters, with minimum inter-cluster distances of between 2.79% and 4.88%. However, BOLD BIN analysis splits one of these clusters (with a maximum intra-cluster distance of 4.27%) into three clusters (*Anopheles rollai* 1, *Anopheles rollai* 2, and *Anopheles rollai* 3, with minimum inter-cluster distances of between 2.01% and 3.76%), thus creating five clusters in total that are herein denoted *Anopheles rollai* 1 through to *Anopheles rollai* 5 (BOLD:ABZ5384, BOLD:AAI4555, BOLD:ADK7866, BOLD:AAI4554, and BOLD:ACR5690, respectively).

*Anopheles neivai* is a highly diverse species complex and is delimited into eight specific clusters using ASAP analyses. Clusters are denoted *An. neivai s.s.* and *An. neivai* 2 through to *An. neivai* 8, with minimum inter-cluster distances ranging from 3.12% (between *An. neivai* 2 and *An. neivai* 3) to 10.09% (between *An. neivai* 3 and *An. neivai* 6), suggesting considerable inter-specific differences. In addition, *An. neivai* 6 has two BINs assigned to it (BOLD:AEL2207 and BOLD:AEH3455), although these are each represented by a single sequence and separated by a distance of just 1.10%. These two BINS were, thus, considered representative of a single taxon (*An. neivai* 6) in the current study. 

There is also some support for a complex structure in *An. homunculus*. Although the ASAP analysis identifies it as a single cluster, the BOLD BIN analysis splits it into two clusters (BOLD:ACB9054 and BOLD:AAF0613) separated by a distance of 2.71% (Figure 2; Table 1). *Anopheles bellator* is also split into two clusters (topotypic *An. bellator* s.s. from Trinidad and Tobago, BOLD:AAJ2798; *An. bellator* 2 from São Paulo, Brazil, BOLD:AAF0614) and separated by an inter-cluster distance of at least 4.11%. *Anopheles laneanus* s.s. (BOLD:AAN3565) is fully resolved across all delimitations. A previously published sequence in GenBank listed as *An. cruzii* (GenBank: KU551284) is herein denoted as *An. laneanus* 2 (BOLD:ADV0367), with which it forms a sister relationship. These are separated by a minimum inter-cluster distance of 3.44%. 

The remaining *An. cruzii* specimens are found as a single cluster (BOLD:AAG3843), although variation within this cluster is high, with a maximum intra-cluster distance of 3.22%. A single specimen (GenBank: KU551285) from the state of the type locality (Rio de Janeiro, Brazil) is found as a sister to the main *An. cruzii* clade in the phylogenetic tree and is separated by a distance of 2.12%. 

## 4. Discussion

**Figure 3:**
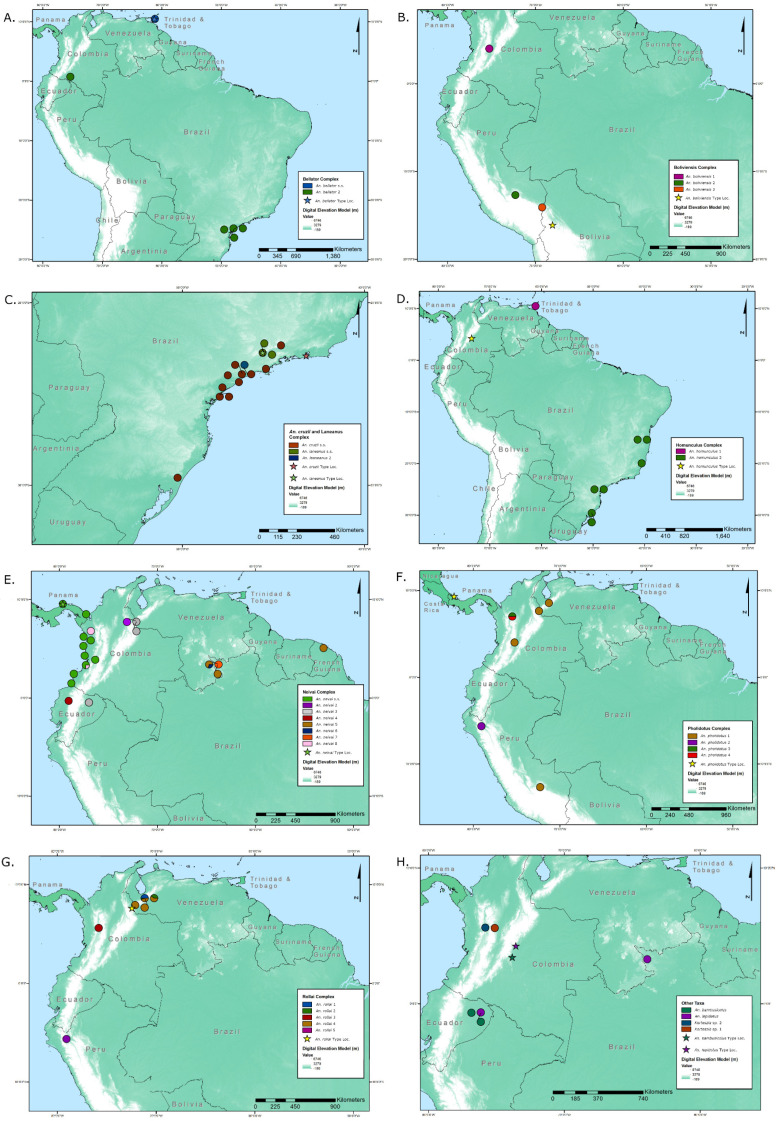
Geographic distribution of subgenus *Kerteszia* clusters from COI species delimitation analyses: (**A**) Bellator complex; (**B**) Boliviensis complex; (**C**) *An. cruzii* and Laneanus complex; (**D**) Homunculus complex; (**E**) Neivai complex; (**F**) Pholidotus complex; (**G**) Rollai complex; (**H**) other *Kerteszia* taxa.

Our combination of phylogenetics and species delimitation analyses of *Kerteszia* mosquitoes reveals high species diversity within this subgenus (Table 1; Figure 1, Figure 2 and Figure 3). 

**Laneanus complex and *Anopheles cruzii*** Previous studies of *An. cruzii* have found that this taxon exists as a species complex. Specimens collected in Brazil from Itatiaia in the state of Rio de Janeiro, and Florianópolis in the state of Santa Catarina, were found to be significantly differentiated at nuclear gene fragments [19]. In addition, chromosomal polymorphism has also been reported, with three forms found among populations from the Brazilian state of São Paulo (Ramirez & Dessen, 2000). More recently, a phylogenetic analysis of the *COI* barcode suggested the existence of three species in the *An. cruzii* complex [17], although genetic distances were not given. Herein, we find cryptic species boundaries between *An. cruzii* and *An. laneanus*. One specimen initially identified as *An. cruzii* (GenBank: KU551284), from the municipality of São Paulo, is clearly resolved from the main *An. cruzii* group, clusters with *An. laneanus* s.s. in both the tree and network and has inter-cluster distances (>3.44%). This level of variation is suggestive of specific differences given that *COI* variation within species of mosquito [36,37,38] and other insects [24,39,40] does not generally exceed 2%. The same sister relationship was found for this specimen and *An. laneanus* s.s. in a previous phylogenetic study using combined COI-ND4 genes, although not with the COI barcode alone [17]. Considering the relationship of this specimen with *An. laneanus* s.s. (which includes specimens from the *An. laneanus* type locality of Campos do Jordão), we have denoted it *An. laneanus* 2, pending further investigation. There is little support for additional species delimitation among the remaining *An. cruzii* specimens, although the maximum intra-cluster distance was higher than expected at 3.22%. The genetic diversity in *An. cruzii* **sensu lato**, its close association with the laneanus complex, and its identity as a potential vector of human pathogens, such as *Plasmodium* [15] and Boraceia virus [41,42], raises important questions about effective approaches to vector incrimination and control in Brazil’s Atlantic Forest. 

**Neivai complex** Specimens identified as *An*. *neivai* showed the highest level of genetic diversity in the current study, with pairwise differences of 14.6% among the most divergent specimens. Species delimitation recovered at least eight tentative species. A basal split within the *An. neivai* clade appears to separate a northern Andean/central American clade from a Guiana Shield clade. Within the former clade, five clusters are identified, two of which (*An. neivai* s.s. and *An. neivai* 3) were found across considerable geographic distances. *Anopheles neivai* s.s. was identified based on its geographical proximately to the type locality of *An. neivai* (~5 km to Portobelo, Panama). The *An. neivai* s.s. cluster retains considerable genetic diversity (maximum intra-cluster distance = 3.15%), mainly due to genetic distances between Panama and Colombia specimens, but these distances were not sufficient to allow for delimitation in our analyses (minimum inter-group distance < 1.20%). This pattern of diversity within *An. neivai* s.s. may represent population differentiation between Panama and Colombia specimens. Within the latter Guiana Shield clade, at least three clusters were identified. These clusters were found in collections made by Silva-do-Nascimento et al. [43] in northwest Roraima State, one of which (*An. neivai* 5) was also collected in the Sinnamary Commune of French Guiana by Talaga et al. [44], demonstrating a geographical distribution that extends for more than 1000 km through much of the Guiana Shield. *Anopheles neivai* is a confirmed vector of important human pathogens. In the Pacific lowlands of Colombia, it has been found to be naturally infected with *Plasmodium falciparum, P. vivax* [45], and Guaroa virus [46], which causes febrile illness in tropical regions of Central and South America [47,48]. It was also found to be infected with yellow fever virus in Panama [49].

The identification of considerable diversity within *An. neivai* s.l. has important implications for vector incrimination in northern South America. *An. neivai* and *An. neivai* 8 (the latter referred to as “*An. neivai* nr. *neivai* 4” in Ahumada et al. [20]) were both found in the department of Valle del Cauca, Colombia, where populations of *An. neivai* s.l. were reportedly infected with Guaroa virus [46]. In addition, specimens of *An. neivai* s.s., collected by López-Rubio et al. [50], were found close (within approximately 40 km) to where historic examples of yellow fever virus-infected *An. neivai* s.l. occurred [49]. Efforts in vector incrimination in the region will, therefore, need to consider the cryptic diversity within *An. neivai* s.l., and further investigate potential differences in epidemiologically and ecologically important traits. 

**Bellator complex***Anopheles bellator* specimens were molecularly partitioned into two tentative species: topotypic *An. bellator* s.s. from Trinidad and Tobago, and *An*. *bellator* 2, which were identified in geographically disparate collections in the Atlantic Forest region of São Paulo, Brazil, and the Amazon region of Putumayo, Colombia, respectively, and separated by distances of approximately 4000 km. *Anopheles bellator* was previously considered an important vector of *Plasmodium* in Trinidad and Tobago, being highly anthropophilic and experimentally susceptible to infection, and it was primarily responsible for the transmission of what became known as “bromeliad malaria” on the islands [9]. In the Atlantic Forest, *An*. *bellator* 2 (as *An. bellator*) reportedly displays considerable endophilic and endophagic behavior [51], although data on *Plasmodium* infection are scant [52].

**Homunculus complex** Although specimens identified as *An*. *homunculus* were not partitioned with ASAP, they were partitioned with the BOLD BIN analyses. However, the maximum intra-specific distance of the ASAP single cluster is 4.1%, and the minimum inter-specific distance of the two clustered proposed by BINs is 2.7% and is suggestive of a species complex structure. In the case of the two clusters of the BIN partition, one is identified from Trinidad and Tobago and the other from Brazil’s Atlantic Forest. Additionally, it appears that the BIN cluster found in Trinidad and Tobago may also be present on the continental mainland. Although not included in the species delimitation analyses, two short COI sequences (307 bp) from two Venezuelan individuals were uniquely invariable with sequences from Trinidad and Tobago (GenBank: OQ272333 and OQ272334). Further sampling and genetic analysis of *An*. *homunculus* specimens, particularly from its type locality of Restrepo, Department of Meta, Colombia, and elsewhere in northern South America, are necessary to clarify the identity of *An*. *homunculus* s.s. and improve our understanding of geographic and evolutionary relationships among island and continental populations. 

**Pholidotus complex** A simple pairwise analysis of all *An*. *pholidotus* specimens found the maximum intra-taxon distance to be considerable (>7%). Our analyses determined that *An*. *pholidotus* was likely a species complex and split this taxon into four clusters. The first was distributed across Colombia, Venezuela, and Peru. The remaining three clusters were each represented by a single specimen; two were collected in the Department of Antioquia in northern Colombia [53], while the third was collected in the Department of Cajamarca in northwestern Peru. Two “Culicidae sp” sequences (GenBank: KY11725 and KY117252; Rosero-García et al. [53]) were also included in this study due to their close identity to *Kerteszia* species following a Blast analysis. Our study allows us to tentatively associate them with the Pholidotus complex. However, without specimens collected from the type locality in northern Panama, we were unable to associate a cluster with the type species. 

***Anopheles lepidotus*** Although the morphological similarity of *An. lepidotus* with *An. pholidotus* has mistakenly implicated the former as a malaria vector in the Department of Tolima, Colombia [26,54], the current study shows that *An. lepidotus* is well resolved as a single cluster in both in tree- and distance-based delimitations and found from Roraima in Brazil to the Province of Orellana in Ecuador, covering almost 1500 km of the Amazon Basin. This cluster may correspond to the type specimen from the Department of Meta, Colombia [25], but molecular data from topotypic *An. lepidotus* are needed to confirm this. The absence of available specimens from the type locality of *An*. *pholidotus* (Bocas del Toro province, Panama) means that we cannot confidently ascribe any cluster as *An. pholidotus* s.s. The potential existence of sibling species in Colombia further complicates our understanding of the competency of these different entities to transmit malaria or other pathogens in the region.

***Anopheles bambusicolus*** Unique among *Kerteszia* species, *Anopheles bambusicolus* is the only species whose larval habitat is found not in bromeliads, but generally in unbroken bamboo internodes. The species’ southern limits are along the Atlantic Forest of southeastern Brazil, while its northern limits are found in the Department of Meta, Colombia, where its type locality exists (La Unión, Department of Meta, Colombia). The specimens included in the current study come from the Province of Orellana, Ecuador, more than 300 km from the type locality, and comprise just a single *COI* haplotype. Further sampling of this species is, thus, required to properly characterize its intra-specific diversity and determine whether this cluster is representative of the type specimen. 

**Boliviensis complex** As can be seen from the polytomy present in the phylogenetic tree, evolutionary relationships among *An. boliviensis* and *An. rollai* are poorly resolved. However, there is strong evidence for both existing as species complexes. *Anopheles boliviensis* 1 was found in Columbia, while *An. boliviensis* 2 and *An. boliviensis* 3 were both collected in the western foothills of the Peruvian Andes, approximately 350 km apart. *Anopheles boliviensis* 1 and *An. boliviensis* 2 form a sister relationship, separated by at least 4.3%, while their evolutionary relationship with *An. boliviensis* 3 is unresolved, and separated by at least 4.6%. We are unable to identify which cluster represents the type species, as our most geographically proximate specimen (*An. boliviensis* 3) was collected more than 250 km from the type locality in Songo, Bolivia. *Anopheles boliviensis* is not considered an important malaria vector, while the importance of orthobunyaviruses Anopheles A and Anopheles B previously found infecting the species remains largely unknown [55]. However, the existence of multiple species within a Boliviensis complex may yet reveal diverse medical importance across its range. 

**Rollai Complex** Two clusters, *An. rollai* 4 and *An. rollai* 5, originating from Venezuela (states of Mérida and Táchira) and Peru (Cajamarca Province), respectively, are consistently resolved. The remaining *An. rollai* specimens are resolved as a single cluster in the ASAP analysis, but split into three clusters in the BOLD BIN analysis, and denoted herein *An. rollai* 1, *An. rollai* 2, and *An. rollai* 3 (BOLD:ABZ5384, BOLD:AAI4555, and BOLD:ADK7866, respectively). Both *An. rollai* 1 and *An. rollai* 2, like *An. rollai* 4, are found in the state of Mérida, Venezuela, while *An. rollai* 3 is found in the department of Antioquia, Colombia. The specimens of *An. rollai* 3 (GenBank: KY117248–KY117250), collected by Rosero-García et al. [53] in Colombia and denoted Culicidae sp. therein, and of *An. rollai* 5, collected by the U.S. Naval Medical Research Unit (NAMRU-6) in Cajamarca state, northern Peru, appear to be new country records for the rollai complex. However, the geographic proximities of *An. rollai* 1, *An. rollai* 2, and *An. rollai* 4 specimens to the *An. rollai* type localities (in the states of Barinas and Táchira, Venezuela) make it difficult to assign clusters to the type species. Biological processes, such as incomplete lineage sorting and introgression, can confound attempts to reconstruct the evolutionary history of closely related species. Given the close evolutionary and geographic relationships among clusters within the Rollai complex, robust phylogenetic inferences will require accounting for such processes using approaches such as the multispecies coalescent model on multi-locus data. Further molecular and morphological analyses of collections at these type localities, as well as from across the Rollai complexes range, will, therefore, be needed to characterize the evolutionary history of the complex and determine which clusters represent the type species. 

Finally, five specimens listed only as Culicidae spp. (GenBank: KY117253–KY117257) collected in Colombia by Rosero-García et al. [53] were included in our study, due to the Blast proximity to *Kerteszia* species. These were shown to comprise two haplotypes recovered in two distinct clusters. Their relationship with other members of *Kerteszia* is difficult to determine due to poor support present in the phylogenetic tree; they are separated from their nearest neighbors by a distance of 4.3% and are herein denoted as *Kerteszia* sp. 

**Missing taxa** Unfortunately, we did not have access to specimens of *An. gonzalezrinconesi* or *An. auyantepuiensis*, both of which are endemic to Venezuela. The inclusion of these two species will be important in future studies to establish their relationships among the diversity described here and to characterize the deeper evolutionary relationships among species in *Kerteszia*. Of course, it remains a possibility that some of the unnamed clusters determined here may indeed correspond to either *An. gonzalezrinconesi* or *An. auyantepuiensis.*

The findings of our study show much higher levels of diversity within the subgenus *Kerteszia* than previously reported. Among the ten currently recognized species that were included in our study, our analyses found at least twenty-eight tentative species present, and some evidence of further cryptic species structure. Previously collected specimens that could only be identified to the level of family are herein denoted members of the subgenus. Despite being able to reveal the existence of several species complexes, *COI* barcode data were unable to resolve deeper evolutionary relationships among species in the subgenus *Kerteszia*. Similarly poor support among these relationships has also been found in the phylogenetic analysis of *Kerteszia* mitogenomes [56]. 

Future work on describing the species diversity and phylogeny of the subgenus will require the analyses of a range of more informative loci, potentially via whole genome sequencing, and using topotypic material where possible. This can allow for assessments of species diversity while accounting for the confounding influences of incomplete lineage sorting and introgression [57,58,59], as well as provide a means to better describe basal nodes and well-supported phylogenetic relationships among members of the subgenus. Furthermore, several of the clusters found, which are tentatively ascribed species, are found as singletons; clearly, further sampling of these clusters is also required to better characterize species diversity within *Kerteszia*. 

## 5. Conclusions

DNA barcoding has proven useful in exposing cryptic biodiversity in mosquitoes. Here, we assessed samples belonging to 10 of the 12 described species in the *Anopheles* subgenus *Kerteszia*. Species delimitation using the distance-based ASAP approach yielded a staggering 28 putative species—almost trebling the currently described taxa in the subgenus. Our findings highlight the importance of using topotypic material to clearly establish the identity of the nominotypical members of complexes, in order to allow formal descriptions of novel species and advocate the use of DNA barcoding approaches in baseline assessments of species diversity, as well as to shape more comprehensive studies of the evolutionary biology of medically important species.

## Figures and Tables

**Table 1:** Type locality information for taxa included in the study.

Species/Taxon	Distribution (by Country)	Type Locality of Sensu Stricto	Type Coordinates		Type Species BOLD BIN	Species Delimitation Clusters	
			Latitude	Longitude		ASAP	BOLD BIN
Neivai complex	Belize, Bolivia, Brazil, Colombia, Costa Rica, Ecuador, El Salvador, French Guiana, Guatemala, Guyana, Mexico, Nicaragua, Panama, Peru, Suriname, Venezuela	Portobelo, Panama	9.554444	−79.655	BOLD:AAN2775	8	9 *
*Anopheles cruzii*	Argentina, Bolivia, Brazil, Colombia, Costa Rica, Ecuador, French Guiana, Guyana, Mexico, Panama, Peru, Suriname, Venezuela	Rio de Janeiro, Brazil	−22.9	−43.2	BOLD:AAG3843	1	1
Laneanus complex	Argentina, Bolivia, Brazil, Peru	Campos do Jordão, São Paulo Brazil	−22.738889	−45.590833	BOLD:AAN3565	2	2
Bellator complex	Brazil, Guyana, Suriname, Trinidad and Tobago, Venezuela	Trinidad and Tobago	10.460556	−61.248611	BOLD:AAJ2798	2	2
Homunculus complex	Bolivia, Brazil, Colombia, French Guiana, Guyana, Peru, Trinidad and Tobago, Venezuela	Restrepo, Meta, Colombia	4.262333	−73.564091	n/a	1	2
Boliviensis complex	Bolivia, Brazil, Colombia, Ecuador, French Guiana, Guyana, Paraguay, Peru, Suriname, Venezuela	Songo, Bolivia	−16.100884	−68.051994	n/a	3	3
Rollai complex	Venezuela	Mata Mulas, Táchira, Venezuela	7.62935	−72.45209	n/a	3	5
		(Unknown locality), Barinas, Venezuela	unknown	unknown			
Pholidotus complex	Bolivia, Colombia, Costa Rica, Ecuador, Panama, Peru, Venezuela	La Zorra, Bocas del Toro, Panama	9.340556	−82.240556	n/a	4	4
*Anopheles bambusicolus*	Argentina, Bolivia, Brazil, Colombia, Ecuador, French Guiana, Guyana, Peru, Suriname, Venezuela	La Unión, Meta, Colombia	3.42787	−73.82803	n/a	1	1
*Anopheles lepidotus*	Bolivia, Colombia, Ecuador, Peru	Restrepo, Meta, Colombia	4.262333	−73.564091	n/a	1	1
Other:							
*Kerteszia* sp.	Colombia	n/a			n/a	2	2
Total						28	32

* Two of these BINS (AEL2207 & AEH3455) were each represented by just a single sequence and separated by a distance of just 1.10%. These were, therefore, considered a single taxon (*An. neivai* 6) in the current study.

## Data Availability

All sequence data used in the study are available on GenBank, with the associated GenBank accession numbers listed in Table S1. These data, and the associated BOLD BIN analysis, are also available on the Barcode of Life Database (BOLD) under Dataset: DS-KERT.

## Supplementary Materials

The following supporting information can be downloaded at: https://www.mdpi.com/article/10.3390/genes14020344/s1, Table S1: Specimen, GenBank and BOLD BIN information.

## Copyright Statement

Some authors are employees of the United States government. This work was prepared as part of their official duties. Title 17 U.S.C. 105 provides that “copyright protection under this title is not available for any work of the United States Government.” Title 17 U.S.C. 101 defines a U.S. Government work as work prepared by a military service member or employee of the U.S. Government as part of that person’s official duties.
